# Do Offspring of Insects Feeding on Defoliation-Resistant Trees Have Better Biological Performance When Exposed to Nutritionally-Imbalanced Food?

**DOI:** 10.3390/insects6010112

**Published:** 2015-01-12

**Authors:** Roberto Quezada-Garcia, Alvaro Fuentealba, Ngoc Nguyen, Éric Bauce

**Affiliations:** 1 Département des sciences du bois et de la forêt, Faculté de foresterie, de géographie et de Géomatique Université Laval, Québec, QC G1V 0A6, Canada; E-Mails: alvaro.fuentealba-morales.1@ulaval.ca (A.F.); Eric.Bauce@vrex.ulaval.ca (E.B.); 2 Department of Biology, Concordia University, 7141 Sherbrooke W., Montreal, QC H4B 1R6, Canada; 3 Direction de l’aménagement et de l’environnement forestiers Ministère des Forêts, de la Faune et des Parcs, 5700, 4^e^ Av. Ouest, Québec, QC G1V 0A6, Canada; E-Mail: ngoc.nguyen@mffp.gouv.qc.ca

**Keywords:** adaptation, *Choristoneura fumiferana*, *Picea glauca*, spruce budworm, tree resistance

## Abstract

White spruce (*Picea glauca* (Moench) Voss) trees that are resistant or susceptible to spruce budworm (*Choristoneura fumiferana* (Clem.)) attack were identified in a southern Quebec plantation. Due to high mortality-induced selective pressures imposed by resistant trees on spruce budworm larvae, insects that survive on resistant trees exhibited greater biological performance than those on susceptible trees. We tested the hypothesis that this better biological performance is maintained across generations when progeny were subjected to nutritional stress. We collected pupae from resistant and susceptible trees (phenotype). Adults were reared under controlled laboratory conditions. Progeny were subsequently reared on two types of artificial diet (high *vs.* low quality). Low quality diet simulated food quality deterioration during outbreak conditions. Results confirmed that surviving insects collected from resistant trees have better performance than those from susceptible trees. Offspring performance (pupal mass, developmental time) was affected only by diet quality. These results suggest that adaptive advantages that would be acquired from parents fed on resistant trees are lost when progeny are exposed to nutritionally-imbalanced food, but the effects persist when larvae are fed a balanced diet. Offspring mortality, fecundity and fertility were positively influenced by parental origin (tree phenotype).

## 1. Introduction

Defense mechanisms in plants that confer resistance to herbivory [1] are closely implicated in the coevolution between insects and plants [2,3]. These mechanisms represent important selection pressures that negatively affect insect performance variables, such as survival, fecundity, pupal mass and growth rate [4]. Insects exposed to selective pressure from resistant hosts may gain adaptations that help maintain or enhance fitness (e.g., survival and fecundity), particularly if the selective stimulus remains constant over multiple generations [5].

Host resistance may differ among tree species, among conspecifics and even within individual trees. This phenomenon can be illustrated with the relationship between spruce budworm and its hosts. The spruce budworm (*Choristoneura fumiferana* (Clemens)) is one of the most important forest pests in boreal North America. Its main host tree is balsam fir, *Abies balsamea* (L.) Miller, followed by white spruce, *Picea glauca* (Moench) Voss, black spruce, *P*. *mariana* (Mill.) BSP, and red spruce, *P*. *rubens* Sargent [6,7]. For example, black spruce is less prone to being defoliated by spruce budworm than balsam fir and white spruce because of late budbreak [6,7] and given that its foliage is rich in toxic compounds that are detrimental to insect performance [8,9]. In contrast, differences between white spruce and balsam fir defoliation has been attributed to the faster growth, greater development and more foliage per unit area in the shoots of spruce compared to those of fir [7].

Differences in host resistance to spruce budworm have also been observed within species. For example, two phenotype of white spruce trees were identified in a plantation near Drummondville, Quebec, Canada (45°53' N, 72°29' W), based on differences in their susceptibility to budworm defoliation [10,11]. Individuals of the first phenotype exhibited less than 25% of defoliation and were classified as resistant to spruce budworm attack, whereas individuals of the second phenotype exhibited more than 60% of defoliation and, therefore, were classified as susceptible [10]. Field rearing tests indicated that spruce budworm larvae fed on foliage of resistant white spruce exhibited high mortality (between 68 and 94%), while larvae fed on foliage of susceptible trees sustained between 30% and 51% mortality. Furthermore, the offspring of parents that had fed on resistant trees had higher survival (90%) than the offspring of parents from susceptible trees (48%) when exposed to foliage of resistant trees. Finally, offspring of parents that had fed on resistant trees exhibited greater biological performance (pupal mass, developmental time and survival) than the offspring of parents that had fed on susceptible trees when reared on artificial diet (McMorran) and foliage from either resistant or susceptible trees [10]. Subsequent studies found that the natural resistance exhibited by some white spruce individuals in this plantation was produced by phenolic compounds that are toxic to spruce budworm [12,13]. Indeed, foliage of resistant white spruce individuals contains higher levels of the acetophenone metabolites, piceol and pungenol, than the foliage of susceptible individuals [12,13], whereas there are no significant differences in nutrient and sugar concentrations [11]. Laboratory assays have shown that the natural concentration of these compounds found in foliage of resistant white spruce can reduce spruce budworm survival up to 50%, as well as reducing pupal mass and increasing developmental time [12]. This may create a selection pressure, eliminating the small, less fit individuals from the population [10].

The greater biological performance exhibited by the offspring of parents from resistant trees could allow this local population to face other selective pressures better than regular populations. This may give an adaptive advantage to this local population to cope with food of low nutritional quality that is found at the end of an outbreak [14,15]. Poor quality food can directly affect the fitness of budworm populations [16], because the insect is very sensitive to nutritional quality [16,17,18]; therefore, it would be interesting to determine if this local population is capable of maintaining this greater performance under nutritionally-stressed conditions. It is important to expand our knowledge on the impact these resistant trees can have on spruce budworm population to be sure that their potential deployment will not increase the severity and duration of insect outbreaks.

The main goal of this study was to determine if offspring of parents that had fed on resistant white spruce trees exhibit greater performance than the offspring of parents that had fed on susceptible trees when reared on a low quality diet that simulates detrimental selection pressures exerted by the low quality food found at the end of an outbreak episode [19]. We hypothesize that nutritionally-stressed conditions will equally affect the performance of the offspring of parents that fed on resistant and susceptible trees. Differences in biological performance between the two sets of offspring, therefore, would be reduced.

## 2. Experimental Section

### 2.1. Study Organism

Spruce budworm pupae were collected from the white spruce experimental plantation that was located in Drummondville, Québec (Canada), during the summer of 2007. Trees from this plantation sustained severe defoliation (more than 60%) by spruce budworm [10,11]. Some resistant individuals were identified within the plantation, which sustained less than 25% defoliation [10]. Based on defoliation percentage, trees were classified as either resistant or susceptible (tree phenotype). In average, there were 16.6 larvae per branch in susceptible trees and 1.85 larvae per branch in resistant trees. Foliage of the resistant phenotype contains higher levels of the acetophenone metabolites, piceol and pungenol, than the foliage of susceptible individuals [12,13], suggesting the presence of constitutive defense mechanisms in the former phenotype [13]. We then collected 700 pupae from resistant trees and 660 pupae from susceptible trees to form the F1 generation, which hereafter is referred to as the parental generation. We assumed that pupae completed their entire life cycle in the trees where they were collected. However, given that we did not use cages, we cannot completely exclude the possibility of some larval dispersal between tree phenotypes. Larvae were sexed and weighed in the laboratory. Adults from each phenotype were subsequently mated (27 pairs from resistant trees; 29 pairs from susceptible trees). Pairs were placed in 11 cm × 7.5 cm cages and moths were fed a 5% sugar-water solution. Eggs were collected every two to three days until the female died. When larvae hatched, they were maintained at 18 °C for two weeks and then placed at 2 °C for 25 weeks (in total darkness) to simulate the winter conditions that were needed for them to complete diapause [20].

Offspring of parents from each tree phenotype were randomly assigned to two different kinds of artificial diets. The first was the standard rearing diet for spruce budworm with 25% sugar and 5% nitrogen content [21]. This diet provides spruce budworm larvae with all of the necessary nutrients required for good performance [19,22] and which hereafter is referred to as the high quality diet. The second diet had lower sugar (1.5%) and higher nitrogen content (7%). This diet induces detrimental effects on larval survival and development that are representative of food quality deterioration during outbreak conditions [19] and which hereafter will be referred to as the low quality diet. All other ingredients, such as minerals, vitamins and water content, remained the same in both diets. Pupae and adults were kept under controlled conditions at 23 °C, 55%–60% relative humidity and a 16 D:8 N photoperiod [20]. Insects were reared in the laboratory in Petri dishes (100 mm × 15 mm), with 10 individuals per dish. Pupae and adults were separated according to sex [20]. Mortality was recorded every day. Couples were installed in circular plastic cages of 11 cm × 7.5 cm. Adults were fed a 5% sugar water solution and allowed to mate. Eggs were collected two or three days after the females died. Substrates that were provided included wax paper for ovipositing and cheese-cloth for overwintering. Larvae that hatched were held at 18 °C for two weeks and were then transferred to 2 °C for 25 weeks to overwinter in dark growth chambers [20].

### 2.2. Biological Parameters

Variables that were measured for the parents were pupal mass (mg), fecundity (total eggs per female) and fertility (egg hatch success rate/total eggs laid). Larval diapause survival (number of live larvae after diapause/total number of larvae in the second instar) and larval developmental time (days) and mortality were measured for the offspring.

### 2.3. Statistical Analyses

Data from the parental generation were subjected to one-way analysis of variance (ANOVA), with tree phenotype (susceptible *vs*. resistant trees) as the treatment. Data from the offspring generation were subjected to two-way ANOVA, with tree phenotype and diet quality (low *vs*. high quality) as the factors using the GLM procedure (SS Type III) of SAS [22]. When data did not satisfy normality and homogeneity assumptions, variables were rank-transformed, and the analysis was performed on ranks. Mortality was analyzed by using logistic regression, assuming the binomial distribution of the response variable, in the GENMOD procedure [22]. Means were compared using Tukey’s adjustments to test for significant interactions.

## 3. Results

### 3.1. Parental Generation

Tree phenotype had a significant effect on the parental generation; more specifically, it affected female and male pupal mass and female fecundity (Table 1). Female and male individuals that originated from resistant white spruce exhibited higher pupal mass (resistant: females, 111.29 ± 1.16 mg; males, 76.99 ± 0.65 mg; susceptible: females, 103.00 ± 1.29 mg; males, 71.79 ± 0.68 mg), but lower fecundity than those coming from susceptible trees (resistant, 292.33 ± 5.61 number of eggs; susceptible, 312.43 ± 5.69 number of eggs).

### 3.2. Offspring

**Figure 1:**
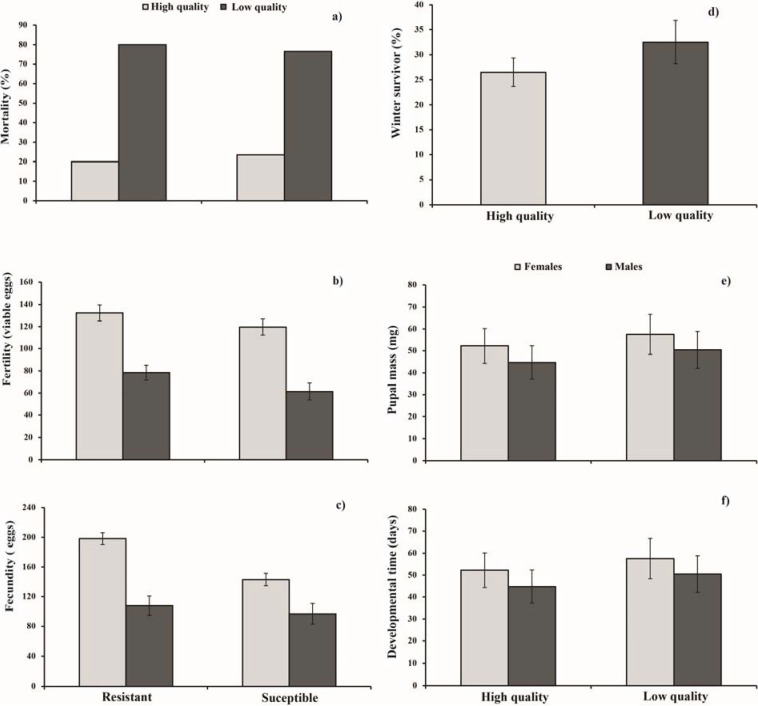
Spruce budworm offspring performance summarized by tree phenotype (**a**–**c**) and diet quality (**d**–**f**) (LSMEANS ± SEM).

Diet quality significantly affected offspring performance. Indeed, pupal mass, developmental time and winter survival were affected by the type of diet (Table 2). Larvae of both sexes that were reared on the high quality diet had higher pupal masses (Figure 1e), shorter developmental times (Figure 1f) and lower mortality (Figure 1a) than those that were reared on the low quality diet. In contrast, overwinter survival was higher on the low compared to the high quality diet (Figure 1d). Tree phenotype had a significant effect on fecundity, fertility and insect mortality (Table 2 and Table 3), while fecundity was also affected by the interaction between tree phenotype and diet (Table 2). Offspring of parents that originated on resistant white spruce exhibited lower mortality (Figure 1a) and higher fertility (Figure 1b) than parents that originated on susceptible white spruce. Furthermore, offspring of parents that originated on resistant white spruce and which were reared on the high quality diet exhibited the highest fecundity. In contrast, offspring of parents that originated on susceptible white spruce and which were reared on low quality diet exhibited the lowest fecundity (Figure 1c).

**Table 1:** One-way ANOVA summaries of the effects of tree phenotype (susceptible *vs*. resistant) on spruce budworm parental generation performance.

	Fertility			Fecundity			Pupal Mass (Females)			Pupal Mass (Males)		
Source of variation	*F*	*df*	*p*	*F*	*df*	*p*	*F*	*df*	*p*	*F*	*df*	*p*
Tree phenotype	0.06	1,351	0.8139	7.51	1,351	0.0064	36.93	1,347	0.0001	50.04	1,365	0.0001

**Table 2:** Two-way ANOVA summaries of the effects tree phenotype (resistant *vs.* susceptible) and diet quality (low *vs*. high) on spruce budworm offspring performance.

	Fecundity			Pupal Mass (Females)			Developmental Time (Females)			Pupal Mass (Males)			Developmental Time (Males)		
Source of variation	*F*	*df*	*p*	*F*	*df*	*p*	*F*	*df*	*p*	*F*	*df*	*p*	*F*	*df*	*p*
Tree phenotype	8.85	1,363	0.0031	2.05	1,363	0.1527	0.14	1,407	0.7085	2.72	1,362	1.000	0.25	1,447	0.6200
Diet	38.12	1,363	0.0001	137.04	1,363	0.0001	25.35	1,407	0.0001	112.40	1,362	0.0001	47.57	1,447	0.0001
Tree phenotype * Diet	3.95	1,363	0.0475	0.90	1,363	0.3438	1.39	1,407	0.2385	0.29	1,362	0.5900	0.00	1,447	0.9850

**Table 3:** ANOVA summaries of the effects of tree phenotype (resistant *vs*. susceptible) and diet quality (low *vs.* high) on spruce budworm offspring fertility, winter survival and mortality.

	Fertility			Winter Survival			Mortality *		
Source of variation	*F*	*df*	*p*	*F*	*df*	*p*	*F*	*df*	*p*
Tree phenotype	4.05	1,353	0.0449	2.94	1,138	0.0885	12.95	1	0.0003
Diet	43.03	1,353	0.0001	7.41	1,138	0.0073	646.06	1	0.0001
Tree phenotype * Diet	0.92	1,353	0.3371	0.13	1,138	0.7169	0.17	1	0.6813

* Mortality was analyzed by using logistic regression, assuming binomial distribution of the response variable, in the GENMOD procedure.

## 4. Discussion

This study reveals that parental generation insects that fed on resistant trees demonstrated higher performance than those fed on susceptible trees. Specifically, pupal mass in both sexes was higher for individuals collected on resistant trees. However, insects from susceptible trees were more fecund than those from resistant trees, but there were no differences in fertility between the two tree phenotypes. These results confirm previous reports that insects that feed on resistant trees have greater biological performance than those that feed on susceptible trees [10]. An early study also found higher pupal mass for insects that originated on resistant trees, from which the authors suggested that high selection pressure exerted by resistant trees eliminates small, less fit individuals [10]. However, the nature of the greater performance observed in larvae fed on foliage of resistant trees is still unknown. Further study is needed to determine if we are in the presence of a genetic adaptation or if the phenomenon is due to epigenetic effects.

Biological parameters of the offspring, such as pupal mass and developmental time, were affected by low quality diet, while tree phenotype influenced none of these parameters. Previous studies have indicated that nutritionally-imbalanced food can affect these variables in spruce budworm [16,23]. Similar responses have been observed in *Malacosoma disstria* (Hübner) [24], *Locusta migratoria* (L.) and *Schistocerca gregaria* (Forskäl) [25], when individuals of these species were reared on an imbalanced diet. These results are not consistent with those of Bauce and Kumbasli [10], who found that offspring of parents fed on resistant trees had greater performance than those fed on susceptible trees, regardless of the food upon which they were reared (artificial diet, foliage, *etc.*). It is noteworthy that these authors, unlike us, used only a high quality diet. The differences in the results between both studies may be therefore explained by the fact that the low quality diet that was used in this study produces a selective pressure so strong, that only the fittest individuals survive. This is confirmed by the mortality that was observed for individuals reared on the low quality diet (Figure 1a). Insects fed on low quality diet had similar performance regardless of parental origin (tree phenotype), which suggests that the greater performance observed in spruce budworm individuals fed on foliage of resistant trees is likely produced by epigenetic effects, since the low quality diet (environment) has the same impact on the offspring of parents from both phenotypes. It may be therefore inferred that individuals from both tree phenotypes exhibit the same capacity to cope with nutritional stresses that are imposed by the diet, simulating a detrimental selection pressure that is incurred by low quality food encountered at the end of an outbreak episode.

Offspring of parents that fed on resistant trees exhibited the highest fecundity and fertility when they were reared on high quality diet. Although not significant, offspring of parents that fed on resistant trees also exhibited greater fecundity and fertility than parents that fed on susceptible trees when reared on low quality food (Figure 1b,c). This result suggests that offspring of parents that fed on resistant trees had a higher capacity for increasing their fecundity and fertility compared to offspring of parents that fed on susceptible trees when reared on good quality food. These life-history traits are highly inheritable [26] and may be implicated in the adaptive process of this forest pest [27]. During outbreak episodes, foliage quantity and quality are significantly reduced by repeated defoliation over several years [28]. This forces the insect to feed on low quality foliage, which has a negative impact on insect performance [29]. If this nutritional stress is endured by several generations, it could have a dramatic effect on the performance of this defoliator [23]. This kind of selection pressure compels the insect to find rapid responses to persist in the environment. Fecundity and fertility may be key factors in their persistence in the environment under stressful conditions [30]. A recent study found that spruce budworm individuals fed on a low quality diet over three generations showed an adaptation in life-history traits, such as fecundity and fertility, to the same low quality diet used in this study [27]. Specifically, larvae that fed on low quality diet over three generations exhibited an increase in the aforementioned life-history traits, from which the authors suggested that spruce budworm may adapt to nutritional stress through maternal effects [27]. Our results show that insects fed on low quality diet exhibited lower fecundity and fertility than those fed on high quality diet, regardless of parental origin. This suggest that the greater performance observed in those individuals that feed on resistant trees does not confer any advantage to them when they face nutritional stress, rendering both populations equally vulnerable to the effects imposed by low quality foliage found at the end of an outbreak episode. Given the results reported by Quezada-Garcia *et al.* [27], the possibility that the insect can adapt to these stressful conditions through maternal effects cannot be discarded. Further research is required to elucidate this issue.

This study has increased our knowledge regarding the relationship between spruce budworm and resistant white spruce individuals. Specifically, our results suggest that insect that can cope with toxic secondary compounds found in the foliage of these resistant individuals do not have any advantage to face nutritional stress and, therefore, may be equally susceptible to low quality food found at the end of an outbreak as insects that feed on susceptible white spruce individuals. Great efforts have been made so far to understand the mechanisms responsible for the natural resistance exhibited by certain white spruce individuals to spruce budworm attack in order to deploy these individuals as methods to reduce the impact of this defoliator. These results strongly suggest that the deployment of these trees would not increase the severity and duration of spruce budworm outbreaks, because even if the insect develops mechanisms to cope with the secondary compounds, they do not seem to be useful for better utilizing low quality food.

## 5. Conclusions

Our results show that differences in biological performance existed between spruce budworm individuals that were collected on resistant and susceptible white spruce. The greater performance exhibited by the parental generation that fed on resistant trees was also observed in its progeny. Yet, exposure to the low quality diet reduced differences in biological performance between the offspring of parents that fed on either resistant or susceptible trees, rendering insects from both tree phenotypes equally susceptible to nutritional stress, similar to the what was found during the decline of an outbreak episode. This suggests that the deployment of resistant white spruce trees would not increase the severity and duration of spruce budworm outbreaks.
